# Supplementary material for: Lung-Derived Selectins Enhance Metastatic Behavior of Triple Negative Breast Cancer Cells
Source: Biomedicines. 2021 Oct 30;9(11):1580. doi: 10.3390/biomedicines9111580 (PMC8615792; doi:10.3390/biomedicines9111580)
Supplement: Supplementary file 1 [file biomedicines-09-01580-s001.zip › biomedicines-1381238-supplementary.pdf]

Supplemental Table S1. Selectin immunodepletion conditions.

Target Selectin	Dynabead® Concentration	Antibody[1] Concentration
E-Selectin (CD62E)	0.6 µg/mL	2 µg/mL
L-Selectin (CD62L)	0.3 µg/mL	4 µg/mL
P-Selectin (CD62P)	0.9 µg/mL	10 µg/mL

[1] All antibodies: polyclonal anti-mouse (R&D Systems)

Supplemental Table S2. Antibody conditions for co-immunoprecipitation and immunoblotting

1° or 2°	Target Protein	Clone	Source	Dilution
1°	Human CD44S	156-3C11	Invitrogen	1:100
2°	Mouse IgG	Polyclonal	Calbiochem	1:2000
1°	Mouse E-Selectin	96403	R&D Systems	1:500
1°	Mouse L-Selectin	95205	R&D Systems	1:1000
2°	Rat IgG	Polyclonal	Calbiochem	1:2000
1°	Mouse P-Selectin	Polyclonal	R&D Systems	1:1000
2°	Goat IgG	Polyclonal	Calbiochem	1:2000

Supplemental Table S3. Media formulations[1] for lung tissue used in the PuMA.

Lung Media 1 (Perfusion)	Lung Media 2 (Culture)
2X M199 Media	1X M199 Media
2.0 µg/mL bovine insulin	1.0 µg/mL bovine insulin
0.2 µg/mL hydrocortisone	0.1 µg/mL hydrocortisone
0.2 µg/mL retinyl acetate	0.1 µg/mL retinyl acetate
200 U/mL penicillin	100 U/mL penicillin
200 µg/mL streptomycin	100 µg/mL streptomycin
7.5% sodium bicarbonate	7.5% sodium bicarbonate

[1] All media and supplements from Invitrogen

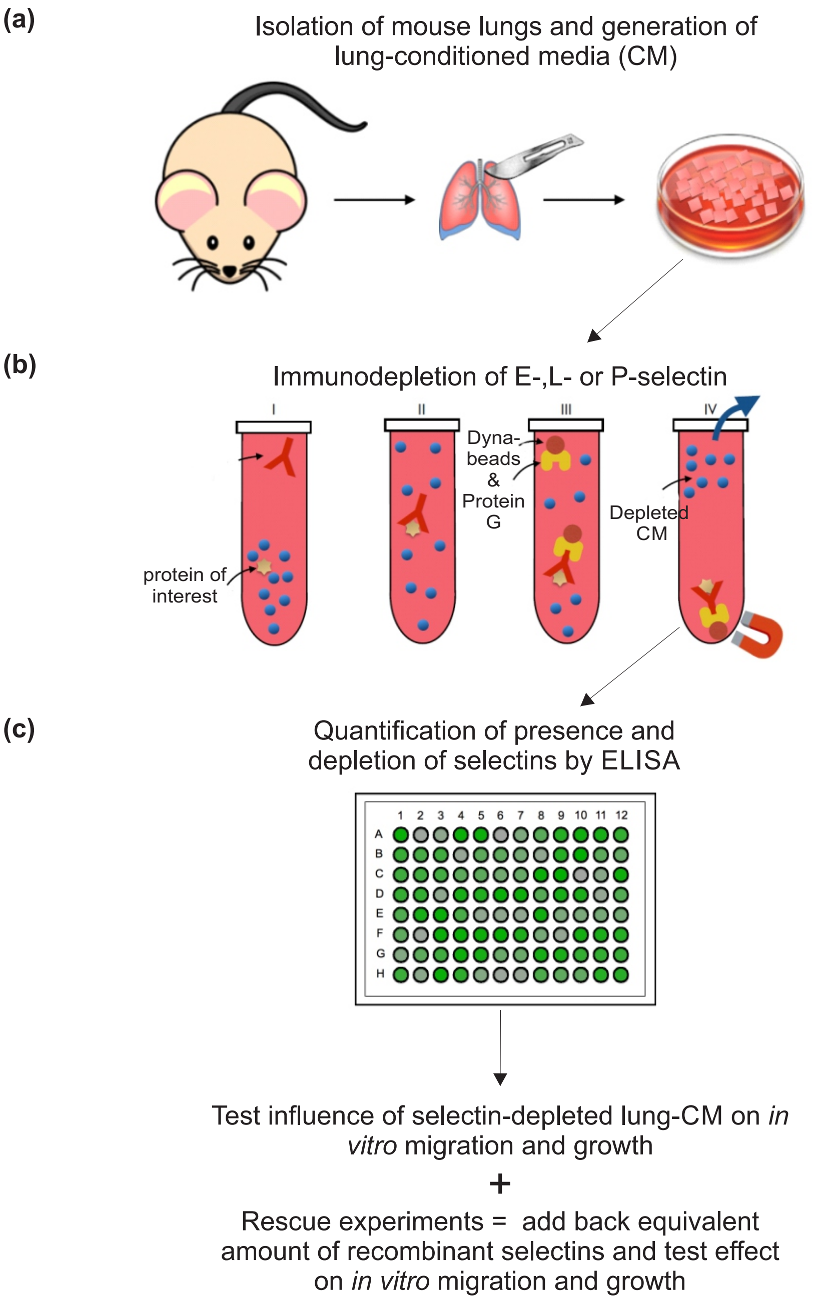

Supplemental Figure S1. Lung-conditioned media (CM) generation and selectin immunodepletion schematic. **(a)** Healthy female nude mice are euthanized by CO_2 inhalation and lungs are aseptically removed. Harvested lungs are washed, minced into ~1 mm^3 fragments, and resuspended in a 4:1 media to tissue (v/w) ratio prior to incubation at 37°C and 5% CO_2. Following a 24 h incubation, lung-CM is harvested, further diluted by three volumes of basal media, centrifuged and filtered prior to use. **(b)** Immunodepletion of E-, L-, or P-selectin from lung-CM is accomplished using magnetic Protein-G Dynabeads® and antibodies specific to soluble mouse selectins as detailed in Table S1. *I.* An antibody specific to one type of selectin (E-, L-, P-) bound to G-protein coupled Dynabeads® is added to lung-CM. *II.* Lung-CM/antibody-Dynabeads® mixture is mixed for 30 min to allow antibodies to bind their protein target present in lung-CM. *III.* After mixing, a magnet is used to remove the selectin antibody/Dynabeads® complexes from lung-CM. *IV.* The remaining lung-CM that is depleted of the target selectin is transferred to a separate tube, filtered through a 0.22 µm syringe filter and stored at -80°C until use for **(c)** ELISA analysis or migration/proliferation assays. For rescue experiments, an equivalent amount of recombinant murine E-, L-, or P-selectin is added back to lung-CM prior to migration and proliferation assays.

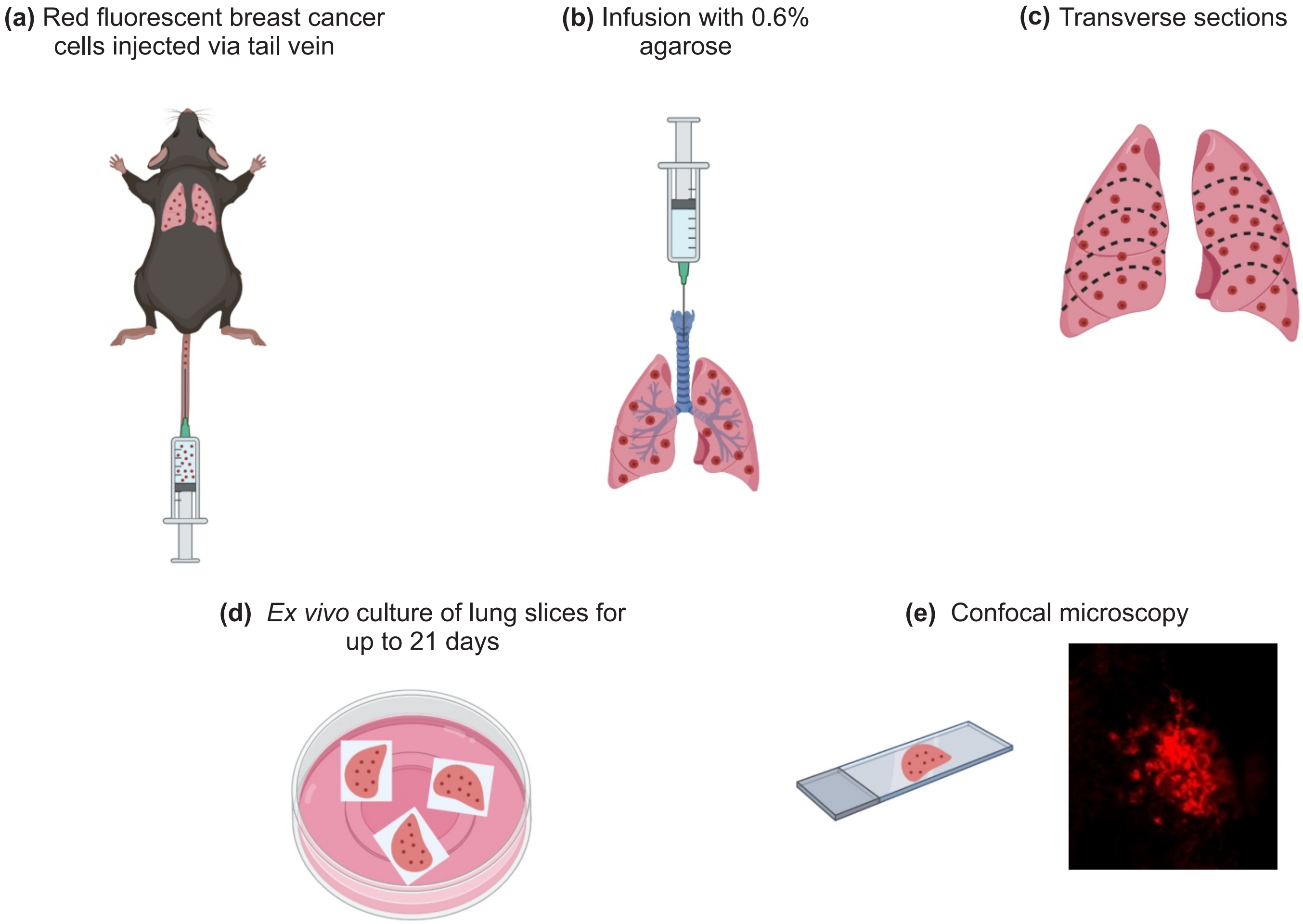

Supplemental Figure S2. Pulmonary metastasis assay (PuMA) schematic. The PuMA offers a real time assessment of breast cancer cell progression from the single cell stage to multicellular colonies in a metastatic setting. **(a)** Female mice are injected tail-vein with red fluorescent breast cancer cells. **(b)** Mice are sacrificed 15 min post injection by Co_2 inhalation. The trachea is carefully cut and cannulated with an 18G blunt needle. The lungs are infused with an agarose/Lung Media 1 (Supplemental Table S2) using gravity perfusion. **(c)** Lungs are carefully removed *en bloc* and cut into ~1 mm transverse sections. **(d)** Lung sections are grown on Gelfoam® pre-incubated with Lung Media 2 (Supplemental Table S2) and grown in culture for up to 21 days. **(e)** Sections are removed from the PuMA at days 0, 7, 14 and 21 and fixed in formalin for 24 h prior to confocal imaging to assess metastatic progression. Schematic created with BioRender.com.
